# Vascular disease in women: comparison of diagnoses in hospital episode statistics and general practice records in England

**DOI:** 10.1186/1471-2288-12-161

**Published:** 2012-10-23

**Authors:** F Lucy Wright, Jane Green, Dexter Canoy, Benjamin J Cairns, Angela Balkwill, Valerie Beral

**Affiliations:** 1 Cancer Epidemiology Unit, University of Oxford, Richard Doll Building, Old Road Campus, Headington, Oxford, OX3 7LF, UK

**Keywords:** Hospital episode statistics, Hospital records, Medical records, Comparison, Diagnosis, Validation, Cohort study, Vascular disease, Myocardial infarction, Stroke, Pulmonary embolism, Venous thromboembolism, Ischaemic heart disease

## Abstract

**Background:**

Electronic linkage to routine administrative datasets, such as the Hospital Episode Statistics (HES) in England, is increasingly used in medical research. Relatively little is known about the reliability of HES diagnostic information for epidemiological studies. In the United Kingdom (UK), general practitioners hold comprehensive records for individuals relating to their primary, secondary and tertiary care. For a random sample of participants in a large UK cohort, we compared vascular disease diagnoses in HES and general practice records to assess agreement between the two sources.

**Methods:**

Million Women Study participants with a HES record of hospital admission with vascular disease (ischaemic heart disease [ICD-10 codes I20-I25], cerebrovascular disease [G45, I60-I69] or venous thromboembolism [I26, I80-I82]) between April 1^st^ 1997 and March 31^st^ 2005 were identified. In each broad diagnostic group and in women with no such HES diagnoses, a random sample of about a thousand women was selected for study. We asked each woman’s general practitioner to provide information on her history of vascular disease and this information was compared with the HES diagnosis record.

**Results:**

Over 90% of study forms sent to general practitioners were returned and 88% of these contained analysable data. For the vast majority of study participants for whom information was available, diagnostic information from general practice and HES records was consistent. Overall, for 93% of women with a HES diagnosis of vascular disease, general practice records agreed with the HES diagnosis; and for 97% of women with no HES diagnosis of vascular disease, the general practitioner had no record of a diagnosis of vascular disease. For severe vascular disease, including myocardial infarction (I21-22), stroke, both overall (I60-64) and by subtype, and pulmonary embolism (I26), HES records appeared to be both reliable and complete.

**Conclusion:**

Hospital admission data in England provide diagnostic information for vascular disease of sufficient reliability for epidemiological analyses.

## Background

Administrative electronic datasets of routinely collected information are increasingly used in medical research. In England, one example is the Hospital Episode Statistics (HES) [1], which contains records of hospital admissions including diagnosis data. The use of such datasets is promoted by the National Institute of Health Research. However, little is known about the reliability of HES diagnostic data for epidemiological studies. General practice records are the most comprehensive source of an individual’s medical history in the United Kingdom (UK), as they include information on investigations and diagnoses in primary, secondary and tertiary care. This study compares the recording of vascular disease diagnoses in HES records with information held by general practitioners for a random sample of participants in a large UK cohort, the Million Women Study. The aim is to assess whether HES diagnoses of vascular disease are of sufficient reliability for epidemiological research.

## Methods

For this study, we used electronic record linkage to identify Million Women Study participants with a HES record of hospital admission with vascular disease (ischaemic heart disease [ICD-10 codes I20-I25], cerebrovascular disease [G45, I60-I69] or venous thromboembolism [I26, I80-I82]) between April 1^st^ 1997 and March 31^st^ 2005. In each broad diagnostic group, a random sample of about a thousand women was selected. For participants with no HES record of the above vascular diseases, a random sample of about a thousand was also selected. We asked each woman’s general practitioner to complete a brief postal questionnaire providing information on her history of vascular disease and this information was compared with the HES diagnosis record.

### Setting: Million Women Study

Between 1996 and 2001, 1.3 million middle-aged women were recruited to the Million Women Study through National Health Service (NHS) Breast Screening Centres in England and Scotland [2]. All study participants gave written consent to follow-up through medical records and approval for the study was obtained from the Oxford and Anglia Multi-Centre Research Ethics Committee. All study participants have a unique NHS number. Using this and other identifying details, they are followed up for deaths, emigration, cancer registrations, changes in name, address and registered general practitioner through electronic linkage with the NHS Central Registers, and for hospital admissions in England through linkage with the HES dataset.

### Data sources

#### Hospital Episode Statistics (HES)

HES is a national administrative dataset of routinely collected individual patient data, containing electronic information on all admissions to NHS hospitals in England. Each admission record includes demographic details, and admission and discharge dates, and consists of one or more consultant episodes (defined as a continuous period of time that a patient spends under the care of a particular consultant). For each episode, coded diagnostic data for the main condition treated or investigated and for any number of other clinical conditions (either pre-existing or occurring during hospitalisation) are recorded using the International Classification of Diseases, 10^th^ revision (ICD-10) [3]. Diagnostic information is extracted from hospital medical notes, coded by trained coders in each hospital and submitted to the central HES data warehouse. It is then prepared for users, such as the NHS, government and researchers. HES information from outpatient settings is limited, and outpatient diagnostic information is currently insufficient for clinical or research use.

#### General practice records

In the UK, general practice records are the most comprehensive source of documentation about an individual’s health and medical care. This reflects general practitioners’ central role in health care delivery in the NHS. All UK residents have the right to be registered with an NHS general practitioner, and rates of non-registration are estimated at less than 0.5% [4,5]. Private (non-NHS) provision accounts for a small proportion of health care in the UK overall and virtually all acute admissions for vascular disease will be through the NHS. General practitioners are the usual first contact for patients seeking non-emergency medical care and initiate virtually all patient referrals to hospital-based specialists in the NHS. General practice records thus cover investigations, treatment and diagnostic information for primary, secondary and tertiary care, including information and documentation on NHS hospital admissions as well as outpatient clinic attendances and general practice consultations. There is currently no complete database of general practice records in England, and so electronic linkage to such records was not feasible for this study.

The Million Women Study includes women from across England and Scotland. Our comparison study was restricted to women who were registered at the time of this study with a general practitioner in selected NHS Comprehensive Local Research Network areas of England, chosen with the aim of ensuring broad geographical coverage. The included areas were: Northumberland, Tyne and Wear; County Durham and Tees Valley; Greater Manchester; Birmingham and the Black Country; Gloucestershire; Thames Valley; Surrey and Sussex; Essex and Hertfordshire; Norfolk and Suffolk.

### Data collection

For this study, HES records were available from April 1^st^ 1997 to March 31^st^ 2005. Three broad groups of vascular disease diagnoses were included in this study: ischaemic heart disease (ICD-10 codes I20-I25), cerebrovascular disease (G45, I60-I69) and venous thromboembolism (I26, I80-I82). For each of the three diagnostic groups, we identified all Million Women Study participants recruited in England who had a relevant HES record within the time period for which HES data were available. In all, 41 982 women with a HES record of ischaemic heart disease, 10 820 with venous thromboembolism and 12 613 with cerebrovascular disease were identified. For each diagnosis group, a random sample of about a thousand women was selected. For each woman, we chose the first HES record after their recruitment to the Million Women Study with the relevant ICD-10 code in any diagnosis field (main or other) as the study admission for comparison with general practice records. From the remaining cohort of women in the Million Women Study with no HES record for vascular disease during the study time period (i.e. none of the above ICD-10 codes after recruitment), a random sample of about a thousand women was also selected.

In June 2010, we wrote to the general practitioner of each woman asking for clinical confirmation of the admission date and diagnosis of vascular disease or not, as recorded in the HES data. Each general practitioner was asked to review their records and complete a one page study form, supply a copy of relevant hospital or other clinical documents, and return these by post. A reminder letter was sent to non-responders after six weeks, and we also wrote to the practice manager of each non-responding general practitioner to ask that they request their general practitioners to complete the study form.

### Data analysis

For each woman selected for this study with a HES record for vascular disease, the ICD-coded diagnoses from HES were compared with clinical diagnoses derived from written information and other documents provided by the general practitioner. Each study form and any documents supplied by general practitioners were reviewed in detail and assigned to a category independently by two cardiovascular researchers with clinical training (FLW & DC). Any disagreements were adjudicated by a panel (FLW, DC, JG, BJC & AB) to reach a consensus. The same review procedure was followed for the study forms and any documents provided by the general practitioner for women with no HES record for vascular disease.

Within each vascular disease group, agreement between HES and general practice records was initially assessed for the broad diagnostic group as a whole. For example, when examining a study form for a HES record of any ischaemic heart disease (I20-I25), the general practice record was classed as consistent for the broad group when the general practitioner reported any diagnosis (confirmed or suspected at the time of admission) within the range of ICD-10 codes of I20-I25. Agreement with general practice records was also assessed for diagnostic subgroups within each broad HES vascular disease group. Within the ischaemic heart disease group, the diagnostic subgroups were myocardial infarction (ICD-10 codes I21-I22) and other ischaemic heart disease (I20, I23-I25). For venous thromboembolism, the subgroups were pulmonary embolism (I26) and venous thrombosis (I80-I82). Within the cerebrovascular disease group, they were transient ischaemic attack (G45), and stroke and other cerebrovascular disorders (I60-I69). To allow examination of stroke subtypes, the cerebrovascular disease group was further split into the following five subgroups: subarachnoid haemorrhage (I60), haemorrhagic stroke (I61-I62), ischaemic stroke (I63), stroke type unspecified (I64) and other cerebrovascular disorders which included stenosis of pre-cerebral or cerebral arteries, (I65-I66), other cerebrovascular diseases (I67-I68) and sequelae of cerebrovascular disease (I69).

General practitioner reports were allocated to one of three categories (Table 1). They were 1) a general practice record with the same diagnosis as the 3 digit ICD-10 diagnosis code in the HES admission, 2) a general practice record with a diagnosis closely related to (i.e. within the same broad diagnostic group as) the 3 digit ICD-10 diagnosis code in the specified HES admission and 3) no general practice record of any vascular disease in the same broad diagnostic group during the study time period. The first two categories included general practitioner reports of diagnoses made either at the same time as or before the HES admission date, since HES records can include pre-existing disease. For the more detailed analyses of cerebrovascular disease, when a general practitioner reported a stroke but of a different type to that in the HES record, the general practice record was classified as having a closely related diagnosis.

**Table 1:** General practitioner report categories for vascular disease* diagnoses in HES records

General practitioner report for selected HES vascular disease* diagnosis:	**Inclusion criteria:**	**Interpretation:**
· General practice record of the same diagnosis as the 3 digit ICD-10 diagnosis code in the HES admission.	· Evidence of the specific diagnosis (confirmed or suspected) at the time of or prior to the HES admission was found in general practice records.	· General practice records agree with the HES record.
· General practice record of a closely related** diagnosis to the 3 digit ICD-10 diagnosis code in the HES admission.	· Evidence of a closely related** diagnosis (confirmed or suspected) at the time of or prior to the HES admission was found in general practice records.	· General practice records broadly agree with the HES record.
· No general practice record of the same or closely related diagnosis as in the HES admission.	· No evidence of any diagnosis within the same broad diagnosis group, at the time of or prior to the specified admission, was found in general practice records; other or no reason apparent for this admission.	· General practice records do not agree with the HES record.

Notes: HES: Hospital Episode Statistics; ICD-10: International Classification of Diseases (10^th^ Revision).

* Ischaemic heart disease: myocardial infarction (ICD-10 codes I21-I22) & other ischaemic heart disease (I20, I23-I25); cerebrovascular disease: transient ischaemic attack (G45) & stroke & other cerebrovascular disorders (I60-I69); and venous thromboembolism: pulmonary embolism (I26) & venous thrombosis (I80-I82.

** within the same broad diagnostic group (e.g. for ischaemic heart disease, a general practice record of myocardial infarction for a HES record of other ischaemic heart disease or vice versa; for cerebrovascular disease, a general practice record of stroke or other cerebrovascular disorders for a HES record of transient ischaemic attack or vice versa; for venous thromboembolism, a general practice record of pulmonary embolism for a HES record of venous thrombosis or vice versa).

For women with no HES record for vascular disease, general practitioner reports were assigned to one of four categories. They were 1) no vascular disease, 2) evidence of ischaemic heart disease, 3) evidence of cerebrovascular disease, and 4) evidence of venous thromboembolism, during the study time period of April 1^st^ 1997 to March 31^st^ 2005. General practice records were classified as consistent with HES when there was no general practice record of a woman having been diagnosed with any vascular disease with or without hospitalisation between April 1^st^ 1997 and March 31^st^ 2005.

All outcomes are reported as proportions with the numbers of women in each general practitioner report category (the numerator) and the total numbers of women with analysable data (the denominator).

## Results

### General practitioners’ return rates and analysable data

Over 90% of study forms sent to general practitioners were returned. Of the returned forms, 88% contained analysable diagnostic data (Table 2). Reasons for unavailable data in returned forms included general practice records being incomplete or no longer available, because the study participant was no longer registered with the practice, or most commonly, she had died. (After a woman’s death, her records are sent to the relevant health authority in the practice region for archiving.) We examined the number of deaths which had occurred before data collection in 2010 in women in each HES diagnostic group, using linked Office for National Statistics mortality data. There were differences in death rates between the women selected because they had a HES record of vascular disease (30% had died by the time of data collection) and those selected because they had no HES record of vascular disease (5% had died). This largely explains the lower return and completion rates from general practitioners for women with a HES record of vascular disease (90% returned, 85% completed with analysable data) compared to those with no such HES record (92% returned, 94% with analysable data) (Table 2). We also examined the proportions of analysable diagnostic data in each diagnostic group by the women’s vital status. For women who were still alive at the time of our data collection, we received analysable diagnostic information for 86% (1892/2120) of women with a HES record of vascular disease and for 87% (829/950) of women with no HES record for any vascular disease. Among those who had died before our data collection, the corresponding figures were 55% (494/892) and 75% (35/47), respectively.

**Table 2:** General practitioner study form return rates and analysable data by HES diagnostic groups

	**HES Diagnosis (ICD-10 code)**			
	**Ischaemic Heart Disease (I20-I25)**	**Venous Thromboembolism (I26, I80-I82)**	**Cerebrovascular Disease (G45, I60-I69)**	**No Vascular Disease**
Study forms sent to general practitioners	N=1004	N=1004	N=1004	N=997
Study forms returned	90.4% (908)	90.3% (907)	90.1% (905)	92.1% (918)
**% of returned study forms with analysable diagnostic data**	**87.7% (796)**	**83.9% (761)**	**84.7% (766)**	**94.1% (864)**

HES: hospital episode statistics; ICD-10: International Classification of Diseases (10th Revision).

### Diagnostic data in HES and general practice records

General practice information was highly consistent with vascular disease diagnostic data recorded in HES (Table 3). Overall agreement was 93% in the three vascular disease diagnostic groups, and 97% in the group with no HES record of no vascular disease.

**Table 3:** Comparison of vascular disease diagnoses in HES and general practice records by HES diagnostic groups

	**HES Diagnosis (ICD-10 codes)**			
	**Ischaemic Heart Disease, (I20-I25)**	**Venous Thromboembolism (I26, I80-I82)**	**Cerebrovascular Disease (G45, I60-I69)**	**No Vascular Disease**
**General Practice Record:**	N=796	N=761	N=766	N=864
Consistent with HES data	91.8% (731)	92.8% (706)	94.0% (720)	97.0% (838)
Differed from HES data	8.2% (65)	7.2% (55)	6.0% (46)	3.0% (26)

HES: hospital episode statistics; ICD-10: International Classification of Diseases (10th Revision).

### Women with a HES record of vascular disease

#### Ischaemic heart disease

For 92% (731/796) of women with an ischaemic heart disease diagnosis (I20-I25) in HES, general practitioners also had a record of ischaemic heart disease, either for the same diagnosis as in the specified admission (88%, 702) or for a closely related ischaemic heart disease diagnosis (4%, 29) (Table 4).

**Table 4:** Comparison of ischaemic heart disease diagnoses in HES and general practice records

	**HES Diagnosis (ICD-10 code)**		
	**Myocardial Infarction (I21-I22)**	**Other Ischaemic Heart Disease (I20, I23-I25**)	**All Ischaemic Heart Disease (I20-I25)**
**General Practitioner Report:**	N=130	N=683	N=796*
Same as HES diagnosis	89.2% (116)	88.1% (602)	88.1% (702)*
Closely related diagnosis	8.5% (11)	2.6% (18)	3.6% (29)
No ischaemic heart disease diagnosis	2.3% (3)	9.2% (63)	8.2% (65)*

HES: hospital episode statistics ; ICD-10: International Classification of Diseases (10th Revision).

* Rows do not total as 17 women with both sub-group ICD-10 codes in selected HES record are included in both sub-group columns.

For women with a HES record of either myocardial infarction (I21-I22) or of other ischaemic heart disease (I20, I23-I25), similar proportions had general practitioner reports of the same diagnosis as in HES (89% and 88%, respectively). For 9% of women with a HES diagnosis of myocardial infarction, general practitioners reported having a record of other ischaemic heart disease. Conversely, for 3% of those with a HES record of other ischaemic heart disease, general practitioners reported a diagnosis of myocardial infarction. The general practitioner reported that an ischaemic heart disease diagnosis had been made prior to the specified admission date for 2% (2/130) of women with a HES record of myocardial infarction, and for 29% (197/683) of women with a HES record of other ischaemic heart disease.

#### Venous thromboembolism

For 93% (706/761) of women with a venous thromboembolism diagnosis (I26, I80-I82) in HES, general practitioners also had a record of venous thromboembolism, with either the same (91%, 693) or a closely related (2%, 13) diagnosis (Table 5). Results were similar for women with a HES record of pulmonary embolism (I26) and women with a HES record of venous thrombosis (I80-I82). Venous thromboembolism diagnoses had been made prior to the specified HES admission date for 4% (10/285) of women with a HES record of pulmonary embolism and 3% (15/495) of women with a HES record of venous thrombosis, according to the general practitioner.

**Table 5:** Comparison of venous thromboembolism diagnoses in HES and general practice records

	**HES Diagnosis (ICD-10 code)**		
	**Pulmonary Embolism (I26)**	**Venous Thrombosis (I80-I82)**	**All Venous Thromboembolism (I26, I80-I82)**
**General Practitioner Report:**	N=285	N=495	N=761*
Same as HES diagnosis	91.2% (260)	91.3% (452)	91.1% (693)*
Closely related diagnosis	1.4% (4)	1.8% (9)	1.7% (13)
No venous thromboembolism diagnosis	7.4% (21)	6.9% (34)	7.2% (55)

HES: hospital episode statistics; ICD-10: International Classification of Diseases (10th Revision).

* Rows do not total as 19 women with both sub-group ICD codes in selected HES record are included in both sub-group columns.

#### Cerebrovascular disease

In the broad cerebrovascular disease diagnostic group (G45, I60-I69), 94% (720/766) of women with a diagnosis in HES had a general practice record either for the same diagnosis as in the specified admission (89%, 681) or for a closely related diagnosis in the broad cerebrovascular disease diagnoses ICD-10 code range (5%, 39). Women with a HES record of transient ischaemic attack (G45) were more likely than those with a diagnosis of stroke or other cerebrovascular disorder (I60-I69) to have a general practice record of a related, rather than the same, diagnosis. For 14% (22/155) of these women, the general practitioner had a record of stroke, whereas a HES record of stroke or other cerebrovascular disorder (I60-I69) was accompanied by a general practice record of transient ischaemic attack for only 3% (17/618). General practitioners reported that a cerebrovascular disease diagnosis had been made prior the specified admission date for 5% (7/155) of women with a HES record of transient ischaemic attack (G45) and 5% (28/618) of women with a HES record of stroke and other cerebrovascular disorders (I60-I69).

Table 6 shows the comparison between HES and general practice records for more detailed diagnostic categories of cerebrovascular disease, including stroke subtypes. Of the 337 women with a HES record of specific stroke subtypes (subarachnoid haemorrhage [I60], haemorrhagic stroke [I61-I62], ischaemic stroke [I63]), 87% (293) had a general practice record for exactly the same stroke type as in HES and another 10% (32) had a general practice record of a stroke but of a different type than in the HES record (classed as a closely related diagnosis). One woman with a HES record of ischaemic stroke had a general practice record of a transient ischaemic attack. For women with a HES record of unspecified stroke (I64), general practitioners reported a record of unspecified stroke for 16% (19/119) and of ischaemic or haemorrhagic stroke for another 74% (88) (categorised as a closely related diagnosis in Table 6). For 3% (3) of these women, general practitioners had a record of transient ischaemic attack. General practitioners reported that the diagnosis had been made prior to the HES admission date for 1% of women with a specific stroke sub-type (4/337) and 1% of those with an unspecified stroke (1/119).

**Table 6:** Detailed comparison of cerebrovascular disease diagnoses in HES and general practice records

		**HES Diagnosis (ICD-10 codes)**						
	**Transient Ischaemic Attack (G45)**	**Stroke**				**Other Cerebrovascular Disorders (I65-I69)**	**All Stroke & Other Cerebrovascular Disorders (I60-I69)**	**All Cerebrovascular Disease (G45, I60-I69)**
		**Subarachnoid Haemorrhage (I60)**	**Haemorrhagic Stroke (I61-I62)**	**Ischaemic Stroke (I63)**	**Unspecified Stroke (I64)**			
**General Practitioner Report:**	N=155	N=78	N=69	N=190	N=119	N=162	N=618	N=766*
Same as HES diagnosis	80.0% (124)	96.1% (75)	78.3% (54)	86.3% (164)	16.0% (19)	82.7% (134)	72.2% (446)	73.8% (565)*
Closely related diagnosis**	14.2% (22)^a^	0	18.9% (13)^b^	10.5% (20)^c^	76.5% (91)^d^	6.8% (11)^e^	21.9% (135)	20.2% (155)*
No cerebrovascular diagnosis	5.8% (9)	3.9% (3)	2.9% (2)	3.2% (6)	7.6% (9)	10.5% (17)	6.0% (37)	6.0% (46)

HES: hospital episode statistics; ICD-10: International Classification of Diseases 10^th^ Revision.

* Rows do not total as 7 women with both G45 and I60-I69 ICD-10 codes in selected HES record are included in both applicable columns.

** Includes general practitioner report of:

^a^ Ischaemic stroke (n=18) & unspecified stroke (n=4).

^b^ Subarachnoid haemorrhage (n=3), ischaemic stroke (n=6) & unspecified stroke (n=4).

^c^ Transient ischaemic attack (n=4), haemorrhagic stroke (n=5) & unspecified stroke (n=11).

^d^ Transient ischaemic attack (n=3), ischaemic stroke (n=78) & haemorrhagic stroke (n=10).

^e^ Transient ischaemic attack (n=11).

Among the 162 women with other cerebrovascular disorders (I65-I69), 83% (134) had a general practice record of the same diagnosis and 7% (11) had a general practitioner report of a transient ischaemic attack*.* Diagnoses had been made before the HES admission for 12% (20/162) of these women according to the general practitioners.

### Women with no HES record of vascular disease

General practice diagnostic information was highly consistent with HES data for women with no HES record of any vascular disease between April 1^st^ 1997 and March 31^st^ 2005. For 97% (838/864) of these women, the general practitioner reported that they had no record of vascular disease for those women during the study time period. Of the remaining 3% (26 women), general practitioners reported that 18 women had a diagnosis of ischaemic heart disease (all with a diagnosis of ischaemic heart disease other than myocardial infarction), six had a cerebrovascular disease diagnosis (three had a transient ischaemic attack and three had suspected stroke) and three had a diagnosis of venous thrombosis without embolism. One woman had been diagnosed with both ischaemic heart disease and venous thrombosis.

For all but one of the 26 women with a general practice vascular disease diagnosis, there was no general practice record of an associated hospital admission. For one woman, the general practitioner reported an admission for stroke, and while there was a HES record for this woman for the relevant date, there was no HES diagnosis code for stroke at that admission. Hospital documents obtained from the general practitioner mentioned a suspected diagnosis of stroke, which was not confirmed by diagnostic imaging. Thus recording of hospital admissions for vascular disease diagnoses in HES records appears to be virtually complete.

## Discussion

### Key findings

For the great majority of participants in our comparison study, diagnostic information in general practice records was consistent with the recording of vascular disease diagnoses in routinely-collected hospital admission (HES) data in England. Overall agreement between the HES record and information from general practice was 93% for women with a recorded hospital admission in the three diagnostic categories (ischaemic heart disease, cerebrovascular disease and venous thromboembolism), and 97% for those with no recorded admission for vascular disease.

Among women with a HES diagnosis of vascular disease, agreement with general practice records was highest for women with a hospital record of myocardial infarction, pulmonary embolism, venous thrombosis and some specific types of stroke. For these women, general practice records agreed with the specific HES diagnosis in around 90% of cases, and with a diagnosis in the same broad diagnostic group in up to 98% of cases. Subarachnoid haemorrhage showed the highest agreement for a specific diagnosis at 96%. For women with an admission diagnosis of transient ischaemic attack (G45) or of cerebrovascular disorders other than stroke (I65-I69), agreement for the specific diagnosis was somewhat lower at around 80%. In women with no HES record of vascular disease, the small numbers of vascular disease diagnoses identified through general practice records were overwhelmingly of less severe and specific disease (no diagnoses of myocardial infarction, confirmed stroke or pulmonary embolism).

### Previous studies

We were unable to find other studies that have compared diagnoses (vascular disease or any other) in HES records with information from general practice records. Diagnoses in routinely collected electronic hospital records have generally been compared directly with hospital medical notes and validated using international diagnostic criteria. A recent systematic review of 25 UK studies published between 1990 and 2010 reported that overall, 80% of coded diagnoses in electronic hospital datasets were confirmed by medical note review; individual study values ranged from 51% to 96%. Only five of these studies included vascular disease in their evaluated diagnostic codes [6]. Validation studies are often difficult to identify through conventional literature searches; in many papers the results of validation exercises may be reported only briefly in the methods or results section, and the name of the dataset used is often not included in the title or keywords. Data providers (including HES) may not keep comprehensive records of studies using their data.

For vascular disease diagnoses, two recently published studies in England found that 100% of myocardial infarction [7] and 96% of haemorrhagic stroke [8] diagnoses were coded correctly in local hospital datasets compared to hospital medical notes. Since 2007 (i.e. after our study period), annual independent audits of HES data have been performed to check the quality of coded data against medical notes in a random sample of 200 records from all English hospitals. A national average of 83% accuracy for all diagnoses (those investigated here, and other vascular and non-vascular diseases) recorded in electronic hospital admission records was reported in 2007/08, which increased to 87% for 2009/10 [9]. Studies from other countries in Europe [10-13] and in North America [14-18] have reported the accuracy of vascular disease diagnoses recorded in hospital datasets as moderate to high, ranging from 69% to 95%.

### Strengths and limitations

This study used random samples from a large cohort of women. We had sufficiently large numbers to examine diagnoses by specific ICD-10 code within the three vascular disease groups. By also sampling women with no record of admission with vascular disease, we were able to assess whether the hospital data were complete and whether the absence of a HES record with a vascular disease diagnosis meant that a woman was free of vascular disease or not during the study time period. The additional information provided by general practitioners about the date of diagnosis allowed us to distinguish a medical history from an acute admission, (a measure of prevalent versus incident disease). Either a medical history of or an acute admission for vascular disease may explain the presence of a diagnostic code in the specified HES record, but they have different implications for epidemiological research.

We had a high return rate from general practitioners of 90%, with 88% of returned forms containing analysable data. General practice information was unavailable for 13% of women with no HES record of vascular disease and 23% of women with a HES record of vascular disease, largely because of differences between the groups in subsequent mortality. We received general practice information for both uncomplicated and complex vascular disease diagnoses, but it is possible that data on uncomplicated diagnoses may have been more likely to be reported to us.

At the time of recruitment to the Million Women Study, study participants represented 1 in 4 of all middle-aged women in England and Scotland and are likely to be reasonably representative of this age group in the general population [19]. Our comparison study included women resident in urban and rural areas across England who were admitted to numerous hospital trusts across the country with responsibility for coding diagnoses for HES. The results are therefore likely to be generalisable to middle-aged women across the NHS in England. However, it is not clear to what extent our results will apply to men, to other age groups in the UK or to other health care settings. A Danish hospital record linkage study found higher accuracy rates for recorded ischaemic heart disease (I20-I22) [10] and deep vein thrombosis (I80) [13] in men compared to women. However, no differences by sex were seen for other vascular disease diagnoses, such as pulmonary embolism (I26) [13] and cerebrovascular diseases (I60-I69) [12].

### Implications for epidemiological research

In this study, HES records were virtually complete for hospital admissions for vascular disease contained in general practice records. For analyses of vascular disease outcomes (identified using broad diagnostic categories) within the Million Women Study, 93% of cases identified through HES records were accurately classified as having a diagnosis of the same or closely related disease. Further, among women with no HES record of vascular disease, 97% were correctly identified. Agreement was highest for the more severe and specific outcomes likely to be of epidemiological interest, such as myocardial infarction, pulmonary embolism, and some specific types of stroke, and for these conditions the great majority of HES records related to diagnoses made at the time of the HES admission. Where the stroke type is specified, HES records also distinguished well between subarachnoid haemorrhage, haemorrhagic and ischaemic strokes.

Not all those with a diagnosis of vascular disease are admitted to hospital. The small proportion of women (26 out of 864; 3%) in our study who had no HES record of vascular disease but did have a clinical diagnosis of vascular disease in general practice records represents a substantial number of non-hospitalised cases. The small numbers of cases involved in our study sample make it difficult to give a precise estimate, but suggest that in the study population there may be around 35,000 non-hospitalised cases of vascular disease in addition to the 61,000 identified through HES. However, the conditions identified in women with no HES record of vascular disease were less severe than those which had led to hospital admission. For example, among the 18 women with a general practice record for ischaemic heart disease but no relevant HES record, none had a diagnosis of myocardial infarction. For the conditions of most interest for epidemiological studies (myocardial infarction, stroke, pulmonary embolism), therefore, HES records appear to capture virtually all cases.

## Conclusion

HES hospital admissions data provide diagnostic information of sufficient reliability and completeness for epidemiological studies of severe vascular disease.

## Abbreviations

HES: hospital episode statistics; UK: United Kingdom; NHS: National Health Service.

## Competing interests

The authors declare that they have no competing interests.

## Authors' contributions

FLW, JG and VB participated in the design of the study. FLW coordinated collection of general practitioner reports. FLW and DC compared general practitioner reports with HES diagnoses. FLW, DC, JG, BJC and AB were members of the review panel. FLW performed the statistical analyses, interpreted the data, and drafted the first version of the manuscript. BJC helped in the statistical analysis and to interpret the data. All authors critically revised the manuscript. All authors read and approved the final manuscript.

## Million Women Study Collaborators

*The Steering Committee are*: Emily Banks, Valerie Beral, Ruth English, Jane Green, Julietta Patnick, Richard Peto, Gillian Reeves, Martin Vessey and Matthew Wallis.

*The Million Women Study Co-ordinating Centre staff are as follows:* Simon Abbott, Naomi Allen, Miranda Armstrong, Angela Balkwill, Emily Banks, Vicky Benson, Valerie Beral, Judith Black, Anna Brown, Diana Bull, Benjamin Cairns, Kathy Callaghan, Karen Canfell, Dexter Canoy, James Chivenga, Barbara Crossley, Francesca Crowe, Dave Ewart, Sarah Ewart, Lee Fletcher, Toral Gathani, Laura Gerrard, Adrian Goodill, Jane Green, Lynden Guiver, Isobel Lingard, Elizabeth Hilton, Sau Wan Kan, Carol Keene, Oksana Kirichek, Mary Kroll, Nicky Langston, Bette Liu, Maria-Jose Luque, Lynn Pank, Kirstin Pirie, Gillian Reeves, Andrew Roddam, Keith Shaw, Emma Sherman, Evie Sherry-Starmer, Helena Strange, Sian Sweetland, Alison Timadjer, Sarah Tipper, Ruth Travis, Xiaosi Wang, Joanna Watson, Lucy Wright, Owen Yang, Heather Young.

*The following NHS Breast Screening Centres took part in the recruitment and breast screening follow up for the Million Women Study*: Avon, Aylesbury, Barnsley, Basingstoke, Bedfordshire and Hertfordshire, Cambridge and Huntingdon, Chelmsford and Colchester, Chester, Cornwall, Crewe, Cumbria, Doncaster, Dorset, East Berkshire, East Cheshire, East Devon, East of Scotland, East Suffolk, East Sussex, Gateshead, Gloucestershire, Great Yarmouth, Hereford and Worcester, Kent, Kings Lynn, Leicestershire, Liverpool, Manchester, Milton Keynes, Newcastle, North Birmingham, North East Scotland, North Lancashire, North Middlesex, North Nottingham, North of Scotland, North Tees, North Yorkshire, Nottingham, Oxford, Portsmouth, Rotherham, Sheffield, Shropshire, Somerset, South Birmingham, South East Scotland, South East Staffordshire, South Derbyshire, South Essex, South Lancashire, South West Scotland, Surrey, Warrington Halton St Helens and Knowsley, Warwickshire Solihull and Coventry, West Berkshire, West Devon, West London, West Suffolk, West Sussex, Wiltshire, Winchester, Wirral, Wycombe.

## Pre-publication history

The pre-publication history for this paper can be accessed here:

http://www.biomedcentral.com/1471-2288/12/161/prepub
